# Reduction of *Klebsiella pneumoniae* bacterial load after active disinfection of venous catheter connectors

**DOI:** 10.1590/0034-7167-2025-0330

**Published:** 2026-11-06

**Authors:** Camila Biazus-Dalcin, Thaís Cristine Marques Sincero, Jefferson Wildes da Silva Moura, Thiago Lopes Silva, Aline de Souza Bitencourt, Isadora Silva de Souza, Denise Miyuki Kusahara, Patrícia Kuerten Rocha

**Affiliations:** I University of Dundee. Dundee, Scotland, United Kingdom; II Universidade Federal de Santa Catarina. Florianópolis, Santa Catarina, Brazil; III Universidade Federal de São Paulo. São Paulo, São Paulo, Brazil

**Keywords:** Bacterial Load, *Klebsiella Pneumoniae;* Catheters, Disinfection, Catheter-Related Infections., Carga Bacteriana, Klebsiella Pneumoniae, Catéteres, Desinfección, Infecciones Relacionadas con Catéteres.

## Abstract

**Objective:**

to verify the effectiveness of active disinfection in reducing the bacterial load of *Klebsiella pneumoniae* inoculated in two types of needleless connectors.

**Method:**

an in vitro experimental study was carried out with 84 experiments. The disinfection treatments used were sterile gauze with 70% ethanol, non-sterile gauze with 70% ethanol, sterile gauze with Incidin^®^, and a single-use device with 70% isopropanol. Data were analyzed using the Kruskal-Wallis test and Conover’s post-hoc test.

**Results:**

all disinfection methods were effective. In valved connectors, the greatest reduction in bacterial load was with 70% ethanol (96.56%), and in extension connectors, the reduction was greater than 99% in all disinfection methods.

**Conclusions:**

the reduction of microbial load in venous access connectors is an important indicator for the prevention of bloodstream infections, in addition to contributing to the promotion of safe practices during nursing care.

## RESUMEN

**Objetivo:**

verificar la eficacia de la desinfección activa para reducir la carga bacteriana de *Klebsiella pneumoniae* inoculada en dos tipos de conectores sin aguja.

**Método:**

estudio experimental in vitro con 84 experimentos. Los tratamientos de desinfección utilizados fueron gasa estéril con etanol al 70%, gasa no estéril con etanol al 70 %, gasa estéril con Incidin^®^ y un dispositivo de un solo uso con isopropanol al 70%. Los datos se analizaron mediante la prueba de Kruskal-Wallis y la prueba post-hoc de Conover.

**Resultados:**

todos los métodos de desinfección fueron efectivos. En los conectores con válvula, la mayor reducción de la carga bacteriana se obtuvo con etanol al 70% (96,56%), y en los conectores de extensión, la reducción fue superior al 99% con todos los métodos de desinfección.

**Conclusiones:**

la reducción de la carga microbiana en los conectores de acceso venoso es un indicador importante para la prevención de infecciones del torrente sanguíneo, además de contribuir a la promoción de prácticas seguras durante la atención de enfermería.

## INTRODUCTION

Bloodstream infection (BSI) is associated with high mortality rates in healthcare services^(1)^. One of the main causes of BSI is the use of vascular access devices (VADs)^(2)^, widely employed in clinical practice for medication administration, parenteral nutrition, and diagnostic procedures. Despite their therapeutic relevance, these devices may promote patient contamination, leading to complications such as infections^(1)^ when not properly used.

A systematic review revealed that approximately one in every 5,606 peripheral intravenous catheters (PIVCs) develops catheter-associated BSI^(3)^. An observational study identified BSI in 15.2% of cases involving central and peripheral VADs across 2,208 bacteremia episodes, mostly in hospital settings^(4)^. Given this scenario, interventions based on protocols that consolidate good care practices are essential, especially those directed toward nursing teams.

It is noteworthy that nursing professionals play a key role in intravenous therapy management, being responsible for VAD maintenance and for the prevention and/or management of complications^(5)^. Inadequate handling of VADs may significantly contribute to the occurrence of catheter-associated BSI^(2)^. In this context, it is essential to understand the causes of catheter-associated BSI and the strategies for its prevention. Contamination may occur via the extraluminal route (external surface of connectors) or intraluminal route (device interior), the latter being associated with biofilm formation. Extraluminal contamination is related to frequent manipulation and prolonged catheter dwell time^(1)^.

Microorganisms, whether pathogenic or not, may contaminate venous connectors such as needleless connectors (NCs), coupled to central vascular access devices (CVADs) and PIVCs^(6)^. These devices are exposed to hospital environments and frequent handling by nursing staff. Therefore, the role of these professionals in maintaining and disinfecting these connectors is emphasized, as they are responsible for administering infused medications and solutions.

In Brazil, a study conducted with NCs used in hospitalized patients revealed contamination in 40% of the analyzed stopcock lumens. In addition, all connectors showed extraluminal bacterial growth. Gram-negative bacilli, such as *Klebsiella pneumoniae*, were identified on 9% of the surfaces of three-way stopcocks^(7)^. *Klebsiella pneumoniae* is a relevant hospital pathogen associated with several healthcare-associated infections (HAIs), such as pneumonia, catheter-associated BSI, wound or surgical site infections, meningitis, septicemia, and urinary tract infections^(8)^. Reports indicate mortality rates between 30% and 70% in HAIs caused by this microorganism^(9)^, with an incidence ranging from 4.43 to 16.43 cases per 1,000 patient-days in Intensive Care Units (ICUs)^(10)^. Furthermore, in Brazil, it is the second most frequent etiological agent in laboratory-confirmed catheter-associated BSI in ICUs^(11)^.

One of the strategies to prevent BSI caused by *Klebsiella pneumoniae* is effective disinfection of NCs, aiming to reduce bacterial load. The disinfection technique must consider the agent used, friction time, and drying time^(2)^. International guidelines recommend the use of chlorhexidine or 70% alcohol-based solutions^(2)^. However, in Brazil, the availability of these disinfectants varies according to the service. Thus, it is essential that nursing teams understand the characteristics of each agent and comply with appropriate friction and drying times to ensure safe care. Despite its importance, many services face shortages of supplies, making proper disinfection practices difficult. Nevertheless, when available, strict adherence to recommendations is essential to ensure process effectiveness.

Moreover, an observational study highlights low adherence to recommendations regarding friction and drying time of disinfectants in clinical practice, which may compromise disinfection effectiveness^(12)^. Although widely addressed in guidelines, there is still heterogeneity in recommendations and gaps regarding controlled experimental assessment of different active disinfection methods, especially considering resources available in the Brazilian context.

Connector disinfection can be classified as active or passive. Active disinfection, known as “scrub the hub”, requires vigorous friction with a disinfectant agent on the NC. Passive disinfection involves the use of protective caps impregnated with disinfectant^(2)^. Although international recommendations prioritize the use of swabs with 70% isopropyl alcohol or alcoholic chlorhexidine for active disinfection, or disinfectant caps for passive disinfection^(2)^, not all Brazilian hospitals have access to these materials. In many settings, the practice is performed using non-sterile gauze (NSG)^(12)^.

In this context, assessing the effectiveness of materials widely used in clinical practice is essential to reflect real working conditions in Brazilian healthcare services. Based on the above, proper disinfection of NCs should be a routine and rigorous nursing practice, as these professionals are primarily responsible for handling connectors during healthcare delivery. Proper disinfection of NCs, using available resources, may contribute to reducing catheter-associated BSI.

### Study relevance

This article is the result of a thesis^(13)^ deposited in the institutional repository: https://repositorio.ufsc.br/handle/123456789/231052.

## OBJECTIVE

To verify the effectiveness of active disinfection in reducing the bacterial load of *Klebsiella pneumoniae* inoculated in two types of NCs.

## METHOD

### Ethical aspects

As this was an *in vitro* laboratory study, review by a Research Ethics Committee involving human subjects was not required.

### Study design

This is an *in vitro* experimental study conducted using the standard strain *Klebsiella pneumoniae* ATCC^®^ 700603™.

### Setting

The study was carried out in a molecular microbiology laboratory in partnership with a research, technology, and innovation laboratory in child and adolescent health, both affiliated with a federal university in southern Brazil.

### Disinfection methods and needleless connectors

Four different disinfection methods were tested on two types of NCs. The tested methods were: I) Sterile gauze (SG) with 70% ethanol (*Mega Química*); II) NSG with 70% ethanol (*Mega Química*); III) SG with Incidin^®^ (Dräger); IV) A single-use device with 70% isopropanol (IPA) (Site-Scrub^®^, Becton Dickinson). The NCs used in the study were a two-way neonatal Luer Lock extension connector (female) (Polifix^®^, B. Braun), used in PIVC, and a valved connector (Safeflow^®^, B. Braun), which can be used in both PIVC and CVAD.

The tested disinfection methods correspond to active disinfection practices of NCs in an inpatient unit of a teaching hospital in southern Brazil. Incidin^®^ was included because it is a product used in the institution. Its application occurred exclusively on the external surfaces of the assessed devices, without direct contact with the catheter lumen or the patient’s bloodstream due to the drying time. It is emphasized that the results presented in this study were obtained exclusively from an *in vitro* experimental model, with no clinical application or recommendation for use in devices that come into direct contact with patients, nor extrapolation beyond the manufacturer’s approved regulatory indications. Additionally, technical triplicates and biological triplicates were performed, along with negative and positive controls (untreated controls), for all experiments. All experiments were conducted under sterile conditions using sterile materials.

### Experimental procedure: standardization, inoculation, treatment, and bacterial quantification

For standardization of the microbial inoculum, the *Klebsiella pneumoniae* strain stored at -20°C was isolated and maintained in Brain Heart Infusion broth, with a maximum growth period of 24 hours. Subculturing was performed for each new biological replicate, and preparation and cultivation followed the recommendations of the Clinical and Laboratory Standards Institute for “Methods for Dilution Antimicrobial Susceptibility Tests for Bacteria that Grow Aerobically”^(14)^. This preparation allowed for counting Colony-Forming Units (CFU). After initial dilution, a 10^-6^ suspension was used, with an average of 15 CFU/mL.

The initial bacterial suspension was prepared with turbidity equivalent to 0.5 McFarland standard (Newprov^®^) and confirmed electronically using the DensiCHEK™ Kit (BioMililux), corresponding to approximately 1.5 × 10^8^ CFU/mL. Subsequently, 1 mL of the working bacterial suspension was prepared by diluting the initial suspension 100-fold (10 μL of the initial *Klebsiella pneumoniae* suspension in 990 μL of sterile 0.9% saline, pipetted into a sterile 15 mL Falcon conical tube). The working suspension contained approximately 1.5 × 10^6^ CFU/mL. The NCs were exposed to the working suspension for five minutes.

It is important to note that all experiments included positive and negative controls. Positive controls (untreated controls) consisted of NCs inoculated with the bacterial suspension and not subjected to any disinfection method, in order to quantify adhered microorganisms.

Negative control NCs were immersed in 1 mL of 0.9% saline for five minutes. For each experimental day, technical triplicates were performed, and biological triplicates were conducted on different days to ensure reproducibility and reliability of the method. Each NC was removed from the sterile Falcon tube using sterile forceps and placed on a sterile, closed Petri dish for drying for 60 minutes in a bacteriological incubator SL-101 (SOLAB) at 35°C ± 1. After drying, each NC was treated with different disinfectants. Treatments followed the manufacturer’s instructions and standard clinical practice recommendations, including those from the Infusion Nursing Society^(2)^, Brazilian National Health Regulatory Agency^(1)^, and Centers for Disease Control and Prevention^(15)^.

Disinfection using SG with 70% ethanol, NSG with 70% ethanol, and SG with Incidin^®^ was performed as follows: 1 mL of disinfectant was pipetted onto the gauze. The extension and valved connectors were removed from the Petri dish using sterile forceps. Friction was then applied to the NCs by rotating the gauze clockwise and counterclockwise for ten seconds (180º). For the extension connector, friction was performed without removing the protective cap. A drying time of five seconds was allowed for both NC types.

For disinfection using the single-use device with 70% IPA, the manufacturer’s instructions were followed, which differed between connector types. For the extension connector, the cap was removed and friction was performed using the device, rotating it clockwise and counterclockwise eight times over ten seconds (180°). For the valved connector, the device was positioned onto the connector, and friction was performed in the same manner, followed by five seconds of drying. All disinfection procedures were performed using the dominant hand of a single researcher, who conducted the *in vitro* experiment, aiming to replicate the same force and technique across all experiments.

It is noteworthy that the five-second drying time adopted in all experiments was chosen because it is more compatible with clinical practice and is supported by evidence demonstrating significant reduction in bacterial contamination within this time. After exposure to disinfection methods, treated samples and controls were placed in 2 mL of 0.9% saline in sterile 15 mL Falcon tubes to allow microorganism recovery. Tubes were sealed with sterile caps. All samples were vortexed using a Phoenix tube shaker (MOD AT56) for five minutes and then subjected to sonication in an ultrasonic bath UltraCleaner 1400 (Unique), at 40 kHz, for ten minutes.

Subsequently, the tubes were vortexed again for five seconds to homogenize the samples. A 100 μL aliquot was plated on Petri dishes containing Tryptic Soy Agar and spread in circular motions using a T-shaped cell spreader (Drigalski) until complete absorption of the inoculum. After incubation for 24 hours at 35°C ± 1 in a bacteriological incubator SL-101 (SOLAB), CFU counting was performed.

### Data analysis and processing

Data analysis involved comparing CFU counts between treated NCs and positive controls. A database was created using Microsoft Excel^®^ 2016 for data storage, organization, and statistical calculations. Two statistical tests were performed: the nonparametric Kruskal-Wallis test, to analyze and compare independent groups, and Conover’s *post-hoc* analysis, to assess interactions between disinfection methods. Median values were used due to asymmetric data distribution, and a significance level of p < 0.05 was adopted. The statistical software used was MedCalc^®^ version 19.1.7 (Belgium).

## RESULTS

A total of 84 experiments were conducted, including 37 with extension connectors and 47 with valved connectors. Overall, 11 positive controls, 12 negative controls, 25 experiments with treatments applied to extension connectors, and 36 experiments with treatments applied to valved connectors were included. It is important to note that outliers were excluded to avoid compromising the experiments, while maintaining at least two technical duplicates and two biological duplicates for validation. No bacterial growth was observed in negative controls, confirming the aseptic conditions of the procedures and materials used.

Among NCs, a median of 377.5 CFU adhered to the positive controls of extension connectors, whereas 236.0 CFU adhered to the positive controls of valved connectors, indicating greater adherence of *Klebsiella pneumoniae* to extension connectors compared to valved connectors. Following the disinfection protocols established in the methodology, all tested methods were effective in reducing bacterial load in both NC types.

In experiments conducted with extension connectors, bacterial load reduction ranged from 69.01% to 96.56% (Table 1). Although variation was observed in reduction rates between treatments using SG and NSG with 70% ethanol, this difference was not statistically significant.

**Table 1:** Effectiveness of active disinfection methods in reducing *Klebsiella pneumoniae* bacterial load in extension connectors. Florianópolis, SC, Brazil, 2025

Active disinfection method	Experiments (n)	Median CFU	Bacterial load reduction (%)	Post-hoc analysis (Conover)^*^ Difference between treatments (p<0.05)
(1) UTC	6	377.5	-	(2)(3)(4)(5)
(2) SG with 70% ethanol	6	117	69.01	(1)
(3) NSG with 70% ethanol	5	13	96.56	(1)
(4) SG with Incidin®	7	108	71.39	(1)
(5) Single-use device with 70% IPA	7	88	76.69	(1)
(6) NC	6	0	-	-

Legend:

* Calculated differences based on the Kruskal-Wallis test and Conover post-hoc analysis with 4 degrees of freedom, with p = 0.002960; UTC - untreated control (positive); NC - negative control; SG - sterile gauze; NSG - non-sterile gauze; IPA - isopropanol; n - total experiments; CFU - Colony-Forming Unit.

Within all treatments applied to extension connectors, the positive control differed significantly from all disinfection treatments. However, no significant differences were observed among the disinfection treatments themselves, despite variations in bacterial reduction percentages (Figure 1). Thus, all treatments proved effective, with a significance level of p = 0.004873.

**Figure 1:**
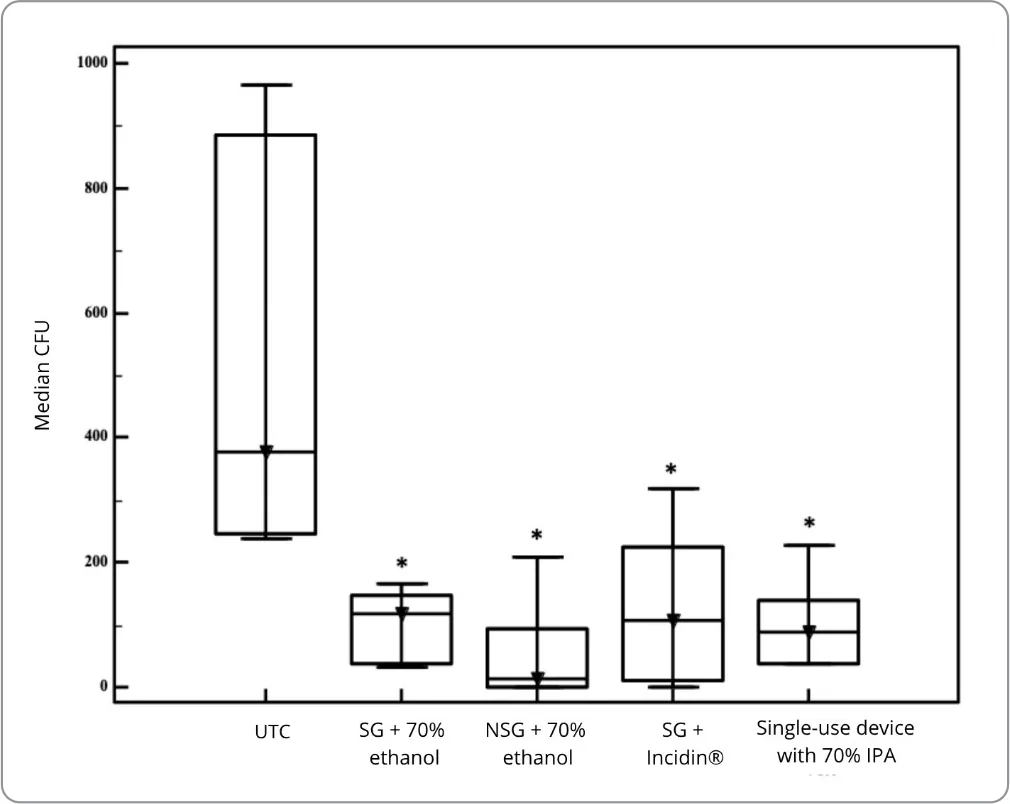
Comparison of the control group with the treatment groups regarding the number of *Klebsiella pneumoniae* Colony-Forming Units in the extension connectors. Florianópolis, SC, Brazil, 2025

Legend: CFU - Colony-Forming Unit; N - total number of experiments; UTC - untreated control (positive); SG - sterile gauze; NSG - non-sterile gauze; IPA - isopropanol; *Different from UTC.

In experiments conducted with valved connectors, a more homogeneous variation in bacterial load reduction was observed among treatments compared to extension connectors. Bacterial load reduction ranged from 99.58% with the single-use device containing 70% IPA to 100% with the other three treatments (p = 0.002960) (Table 2).

**Table 2:** Effectiveness of active disinfection methods in reducing *Klebsiella pneumoniae* bacterial load in valved connectors. Florianópolis, SC, Brazil, 2025

Active disinfection method	Experiments (n)	Median CFU	Bacterial load reduction (%)	Post-hoc analysis (Conover)^*^ Difference between treatments (p<0.05)
(1) UTC	5	236	-	(2)(3)(4)(5)
(2) SG with 70% ethanol	9	0	100	(1)
(3) NSG with 70% etanol	9	0	100	(1)
(4) SG with Incidin^®^	9	0	100	(1)
(5) Single-use device with 70% IPA	9	1	99.58	(1)
(6) NC	6	0	-	-

Legend:

* Calculated differences based on the Kruskal-Wallis test and Conover post-hoc analysis with 4 degrees of freedom, with p = 0.002960; UTC - untreated control (positive); NC - negative control; SG - sterile gauze; NSG - non-sterile gauze; IPA - isopropanol; n - total experiments; CFU - Colony-Forming Unit.

Figure 2 shows the comparison among experiments conducted with valved connectors. All treatments resulted in reduced bacterial load compared to the positive control. Greater variability was observed in the quartile distribution for the single-use device with 70% IPA; however, this did not affect the overall reduction in bacterial load compared to the untreated group. All treatments were effective in reducing bacterial load and showed no significant differences among them.

**Figure 2:**
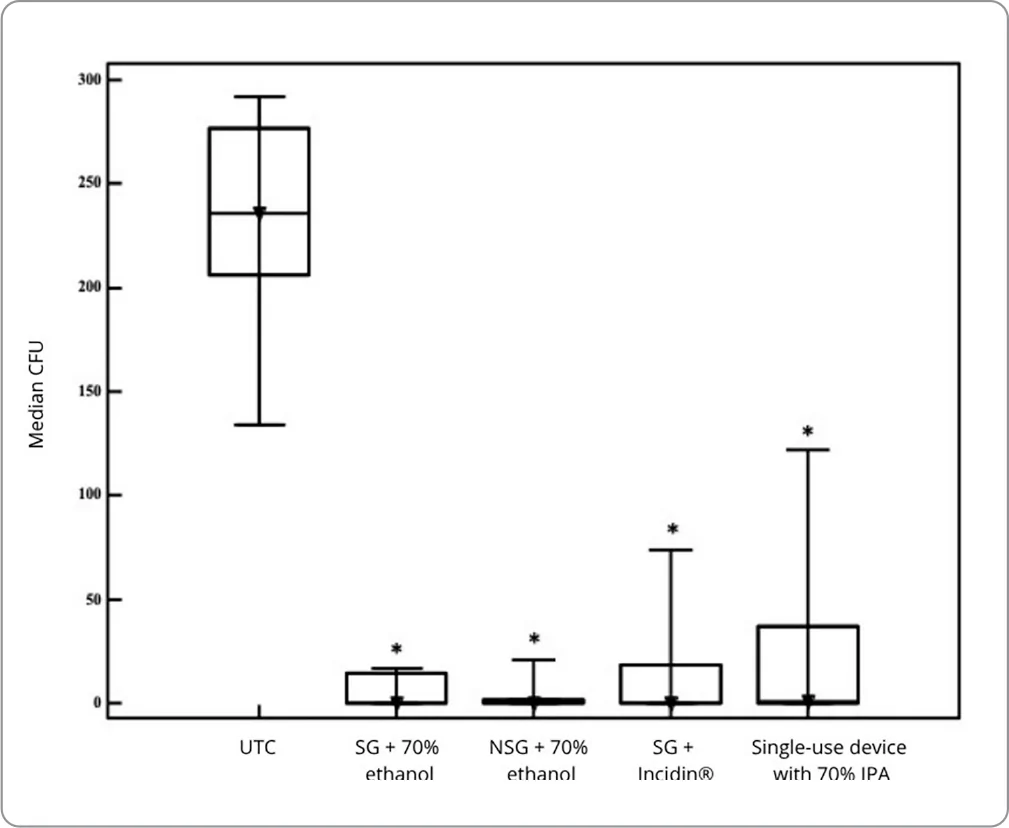
Comparison of the control group with the treatment groups regarding the reduction in the number of *Klebsiella pneumoniae* Colony-Forming Units in the valved connectors. Florianópolis, SC, Brazil, 2025

Legend: CFU - Colony-Forming Unit; N - total number of experiments; UTC - untreated control (positive); SG - sterile gauze; NSG - non-sterile gauze; IPA - isopropanol; *Different from UTC.

## DISCUSSION

The effectiveness of the four active chemical disinfection methods in reducing the bacterial load of *Klebsiella pneumoniae* inoculated in extension and valved connectors was demonstrated. All treatments showed statistically significant reductions in CFU, indicating potential for mitigating the risk of catheter-associated BSI.

In this study, NC disinfection was effective using 70% ethanol, Incidin®, and 70% IPA. A randomized clinical trial (RCT) comparing other disinfection methods in valved connectors showed no difference in effectiveness between wipes containing 70% IPA and wipes containing 2% chlorhexidine gluconate (CHG) with 70% IPA for NC disinfection. Due to their low allergy risk and low cost, the use of 70% IPA for a minimum friction time of five seconds was recommended^(16)^.

Conversely, another study suggests greater effectiveness of active disinfection with 2% CHG compared to 70% IPA^(17)^. International guidelines have evolved with emerging evidence; while earlier recommendations included povidone-iodine among the options^(18)^, this recommendation has since been excluded due to its prolonged drying time (more than six minutes)^(2)^.

It is important to highlight that gauze was used in this study to reflect the reality of many national hospitals. Both SG and NSG were used, mirroring daily nursing practice. The NSG used in this study was stored in clean environments, as commonly occurs in hospital units. A cross-sectional study conducted in inpatient units of a pediatric hospital in Brazil showed that 83.7% of disinfection procedures were performed using NSG^(12)^. However, in countries such as the United States, Australia, and European nations, there is predominant use of wipes with 70% IPA, IPA caps, wipes with 2% CHG, and wipes with 70% IPA^(19)^.

This study assessed the adherence of *Klebsiella pneumoniae* ATCC^®^ 700603™ to the different NCs tested. Greater adherence was observed in extension connectors compared to valved connectors. This suggests that there may be differences in the risk of catheter-associated BSI among different NC types, potentially related to connector design, disinfection method, and manufacturing processes^(20)^, which could be a hypothesis for the finding.

A study that examined catheter-associated BSI in relation to Central Venous Catheter (CVC) use and different NCs found that infection risk was higher when patients used three-way stopcocks^(21)^. It is known that stopcocks have an external design similar to the extension connectors used in this experiment. On the other hand, an RCT conducted with ICU surgical patients showed that NC type did not influence CVC-associated BSI rates. In that study, 23 patients in the control group used stopcocks, while 24 patients in the intervention group used valved connectors^(22)^.

Previous studies have typically compared three-way stopcocks and valved connectors^(21,23)^, due to their more frequent use in CVAD. However, this study presents a comparison between extension connectors-commonly used in the Brazilian hospital setting for PIVC-and valved connectors, which are also used in PIVC but more frequently in CVAD.

In this study, friction was performed for ten seconds, which was sufficient to reduce bacterial load. In all experiments, a drying time of five seconds was allowed. Previous studies have demonstrated the need for friction times greater than three seconds at the intravenous infusion port to significantly reduce bacterial contamination^(23)^. Another study showed that friction was effective at durations of five, ten, and 15 seconds^(16)^.

Current guidelines recommend friction for five to 15 seconds and drying for five seconds when using 70% IPA^(2)^. Therefore, friction times longer than five seconds are already effective for reducing bacterial load and should be considered alongside other disinfection factors, such as disinfectant type, NC type, friction technique applied by nursing professionals, and drying time.

A scoping review that aimed to identify NC disinfection methods and their effectiveness reinforces the importance of nursing professionals understanding disinfectant agents as well as friction and drying times. However, despite the availability of multiple methods, there is still no consensus on the best approach for clinical practice^(24)^.

A retrospective study conducted in a renal transplant unit, involving 900 transplants in recent years, identified 86 cases of carbapenem-resistant *Klebsiella pneumoniae* infections, with BSI being the most frequent site and an overall mortality rate of 36% within 30 days^(25)^.

Another retrospective documentary study analyzing medical records of patients with positive cultures for *Klebsiella pneumoniae* found that CVAD use was a risk factor for infection development, present in 18.8% of cases, including deaths resulting from infection^(26)^. The increasing resistance of *Klebsiella pneumoniae* to antibiotics is concerning^(25,26)^, requiring healthcare institutions to adopt measures to reduce infections caused by this bacillus. Disinfection of venous devices used in intravenous therapy is an important action to be performed by the nursing team.

Within the healthcare workforce, nursing professionals-being responsible for maintaining venous access and, consequently, NCs-must remain attentive to care-related actions that may lead to adverse events. The lack of scientific evidence, insufficient knowledge among nurses regarding catheter-related practices, and weaknesses in professional training related to infection control represent risks to patient safety^(27-29)^.

Disinfection is recognized as one of the steps for the prevention and control of catheter-associated BSI caused by *Klebsiella pneumoniae* ^(30)^. Healthcare professionals must follow infection prevention protocols, including hand hygiene before and after any procedure and proper disinfection of surfaces that come into contact with patients, such as NCs^(31)^. Therefore, the need for specific protocols for the prevention of catheter-associated BSI-both peripheral and central-is evident, aiming to reduce mortality and complications related to healthcare delivery.

### Study limitations

A limitation of this study is the use of only one bacterial strain for contamination of venous access connectors, which does not fully reflect clinical practice. The use of a strain from a bacterial repository allowed greater experimental control and reproducibility; however, it does not fully represent the behavior of multidrug-resistant clinical strains, constituting an additional limitation. Furthermore, sample size may have influenced the results due to the exclusion of outliers.

### Contributions to nursing and health

This *in vitro* study represents an initial step toward understanding *Klebsiella pneumoniae*’s behavior in response to disinfectants available in Brazilian hospitals. It is essential that nursing professionals incorporate this knowledge so that their clinical practice is grounded in scientific evidence, ensuring safe patient care without adverse events related to intravenous therapy. Additionally, the findings support the planning of health education and continuing education initiatives aimed at disseminating and updating nursing knowledge regarding the importance of NC disinfection, as well as appropriate friction and drying times for each product, thereby ensuring process effectiveness.

## CONCLUSION

All disinfection methods (treatments) were effective in reducing the bacterial load of *Klebsiella pneumoniae* ATCC^®^ 700603™ inoculated in *in vitro* experiments. No statistical difference was observed among the different active disinfection methods (SG with 70% ethanol, NSG with 70% ethanol, SG with Incidin^®^, and single-use device with 70% IPA) when applied to extension and valved connectors. Therefore, the results demonstrate that friction for ten seconds using one of the three disinfectant solutions (70% ethanol, Incidin^®^, or 70% IPA), followed by a five-second drying time of NCs, is effective in reducing the bacterial load of *Klebsiella pneumoniae.*

These findings are relevant for nursing clinical practice, as they identify effective NC disinfection methods that may contribute to reducing catheter-associated BSI rates in hospitals for patients using PIVC and CVAD. This represents a first step toward identifying safe disinfection practices for NCs and should be followed by RCTs to confirm experimental findings and support protocol development for healthcare professionals.

It is important to conduct further experiments using SG and NSG to verify differences between these materials, even though they were considered a non-significant experimental variation in this study. Additionally, cost-benefit considerations should be taken into account when selecting disinfectants and disinfection methods in Brazilian hospitals.

## Data Availability

Available research data is in the body of the article.
