# Supplementary material for: Reduction of Klebsiella pneumoniae bacterial load after active disinfection of venous catheter connectors
Source: Rev Bras Enferm. 2026 Nov 6;79:e20250330. doi: 10.1590/0034-7167-2025-0330 (PMC13743047; doi:10.1590/0034-7167-2025-0330)
Supplement: Translation

# Translation: Redução da carga bacteriana de *Klebsiella pneumoniae* após desinfecção ativa de conectores de cateteres venosos

**DOI:** 10.1590/0034-7167-2025-0330pt

**Authors:** Camila Biazus-Dalcin, Thaís Cristine Marques Sincero, Jefferson Wildes da Silva Moura, Thiago Lopes Silva, Aline de Souza Bitencourt, Isadora Silva de Souza, Denise Miyuki Kusahara, Patrícia Kuerten Rocha

**Affiliations:** I University of Dundee. Dundee, Escócia, Reino Unido; II Universidade Federal de Santa Catarina. Florianópolis, Santa Catarina, Brasil; III Universidade Federal de São Paulo. São Paulo, São Paulo, Brasil

**Keywords:** Carga Bacteriana, *Klebsiella Pneumoniae;* Cateteres, Desinfecção, Infecções Relacionadas a Cateter.

## Abstract

**Objetivo:**

verificar a eficácia de desinfecção ativa para a redução da carga bacteriana da *Klebsiella pneumoniae* inoculada em dois tipos de conectores sem agulha.

**Método:**

estudo experimental, *in vitro*, com 84 experimentos. Os tratamentos de desinfecção utilizados foram gaze estéril com etanol 70%, gaze não estéril com etanol 70%, gaze estéril com Incidin^®^ e dispositivo de uso único com isopropanol 70%. Os dados foram analisados pelos testes de Kruskal-Wallis e post-hoc de Conover.

**Resultados:**

todos os métodos de desinfecção foram eficazes. Em conectores valvulados, a maior redução de carga bacteriana foi com etanol 70% (96,56%), e nos conectores de extensão, a redução foi superior a 99% em todos os métodos de desinfecção.

**Conclusões:**

a redução da carga microbiana em conectores de acessos venosos é um importante indicativo para a prevenção de infecções da corrente sanguínea, além de contribuir para a promoção de práticas seguras durante a assistência de enfermagem.

## INTRODUÇÃO

A infecção de corrente sanguínea (ICS) está associada a altas taxas de mortalidade nos serviços de saúde^(1)^. Uma das principais causas de ICS é o uso de dispositivos de acesso vascular (DAVs)^(2)^, amplamente empregados na prática clínica para administração de medicamentos, nutrição parenteral e realização de exames. Apesar de sua relevância terapêutica, esses dispositivos podem favorecer a contaminação do paciente, levando a complicações, como infecções^(1)^, quando não utilizados adequadamente.

Revisão sistemática revelou que aproximadamente um em cada 5.606 cateteres intravenosos periféricos (CIVPs) desenvolve ICS associada ao cateter^(3)^. Estudo observacional identificou ICS em 15,2% dos casos de DAVs centrais e periféricos em 2.208 episódios de bacteremia, majoritariamente em ambiente hospitalar^(4)^. Diante desse cenário, são imprescindíveis intervenções baseadas em protocolos que consolidem boas práticas assistenciais, especialmente voltadas à equipe de enfermagem.

Cabe destacar que os profissionais de enfermagem desempenham papel essencial na gestão da terapia intravenosa (TIV), sendo responsáveis pela manutenção dos DAVs e pela prevenção e/ou manejo de complicações^(5)^. A manipulação inadequada dos DAVs pode contribuir significativamente para o surgimento de ICS associada ao cateter^(2)^. No cenário em tela, é fundamental compreender as causas da ICS associada ao cateter e as estratégias para sua prevenção. A contaminação pode ocorrer por via extraluminal (superfície externa dos conectores) ou intraluminal (interior do dispositivo), sendo esta última associada à formação de biofilme. Já a contaminação extraluminal está relacionada à manipulação frequente e à permanência prolongada do cateter^(1)^.

Microrganismos, patogênicos ou não, podem contaminar conectores venosos, como os conectores sem agulha (CSAs), acoplados a dispositivos de acesso venoso central (DAVCs) e CIVPs^(6)^. Esses dispositivos ficam expostos ao ambiente hospitalar e ao manejo frequente da equipe de enfermagem. Destaca-se, portanto, o papel desses profissionais na manutenção e na desinfecção desses conectores, uma vez que são responsáveis pela administração de medicamentos e soluções infundidas.

No Brasil, estudo realizado com CSAs utilizados em pacientes hospitalizados revelou contaminação em 40% dos lúmens das dânulas analisadas. Além disso, todos os conectores apresentaram crescimento bacteriano extraluminal. Em 9% das superfícies das dânulas de três vias, foram identificados bacilos Gram-negativos, como *Klebsiella pneumoniae* ^(7)^. A *Klebsiella pneumoniae* é um patógeno hospitalar relevante, associado a diversas infecções relacionadas à assistência à saúde (IRAS), como pneumonia, ICS associada ao cateter, infecções de feridas ou sítio cirúrgico, meningite, septicemia e infecção do trato urinário^(8)^. Relatos apontam mortalidade entre 30% e 70% em IRAS causadas por esse microrganismo^(9)^, com incidência de 4,43 a 16,43 casos por 1.000 pacientes-dia em Unidade de Terapia Intensiva (UTI)^(10)^. Além disso, no Brasil, é o segundo agente etiológico mais frequente em ICS associada ao cateter confirmada laboratorialmente em UTIs^(11)^.

Uma das estratégias para prevenir ICS por *Klebsiella pneumoniae* é a desinfecção eficaz dos CSAs, visando à redução da carga bacteriana. A técnica de desinfecção deve considerar o agente utilizado, o tempo de fricção e o tempo de secagem^(2)^. Diretrizes internacionais recomendam o uso de clorexidina ou soluções à base de álcool a 70%^(2)^. Contudo, no Brasil, a disponibilidade desses desinfetantes varia conforme o serviço. Assim, é essencial que a equipe de enfermagem conheça as características de cada agente e respeitem o tempo adequado de fricção e secagem, a fim de garantir um cuidado seguro. Apesar de sua importância, a realidade de muitos serviços inclui escassez de insumos, dificultando a prática adequada da desinfecção. Ainda assim, quando disponíveis, é imprescindível seguir rigorosamente as recomendações de uso para assegurar a eficácia do processo.

Além disso, estudo observacional evidencia baixa adesão às recomendações quanto ao tempo de fricção e ao tempo de secagem dos desinfetantes na prática clínica, o que pode comprometer a eficácia do processo de desinfecção^(12)^. Embora o tema seja amplamente abordado em diretrizes, ainda há heterogeneidade nas recomendações e lacunas quanto à avaliação experimental controlada de diferentes métodos de desinfecção ativa, especialmente considerando os recursos disponíveis na realidade brasileira.

A desinfecção dos conectores pode ser classificada como ativa ou passiva. A desinfecção ativa, conhecida como “*scrub the hub*”, exige fricção vigorosa com agente desinfetante no CSA. Já a desinfecção passiva envolve o uso de tampas protetoras embebidas com desinfetante^(2)^. Apesar de as recomendações internacionais priorizarem o uso do *swab* com álcool isopropílico a 70% ou clorexidina alcoólica na desinfecção ativa, ou tampas protetoras com desinfetante na desinfecção passiva^(2)^, nem todos os hospitais brasileiros dispõem desses materiais. Em muitos locais, a prática é realizada com gaze não estéril (GNE)^(12)^.

Nesse contexto, avaliar a eficácia de materiais amplamente utilizados na prática assistencial é fundamental para refletir as condições reais de trabalho nos serviços de saúde brasileiros. Mediante o exposto, a desinfecção adequada dos CSAs deve ser uma prática rotineira e criteriosa da enfermagem, visto que esses profissionais são os principais responsáveis pela manipulação dos conectores durante a assistência à saúde. A correta desinfecção dos CSAs, a partir dos recursos disponíveis, pode contribuir para a redução de ICS associada ao cateter.

### Relevância do estudo

Este artigo é produto de tese^(13)^ depositada no repositório institucional: https://repositorio.ufsc.br/handle/123456789/231052.

## OBJETIVO

Verificar a eficácia de desinfecção ativa para a redução da carga bacteriana da *Klebsiella pneumoniae* inoculada em dois tipos de CSAs

## MÉTODOS

### Aspectos éticos

Por se tratar de estudo *in vitro*, realizado em laboratório, foi dispensada a apreciação pelo Comitê de Ética em Pesquisa Envolvendo Seres Humanos.

### Desenho do estudo

Estudo experimental realizado *in vitro* com a cepa padrão *Klebsiella pneumoniae* ATCC^®^ 700603™.

### Local

O estudo foi realizado em um Laboratório de Microbiologia Molecular Aplicada em parceria com um Laboratório de Pesquisa, Tecnologia e Inovação na Saúde da Criança e do Adolescente, ambos vinculados a uma Universidade Federal de um estado do sul do Brasil.

### Métodos de desinfecção utilizados e conectores sem agulhas

Foram testados quatro métodos diferentes de desinfecção em dois tipos de CSAs. Os métodos de desinfecção testados foram: I) Gaze estéril (GE) com etanol 70% (Mega Química); II) GNE com etanol 70% (Mega Química); III) GE com Incidin^®^ (Dräger); e IV) Dispositivo de uso único com isopropanol (IPA) 70% (Site-Scrub^®^, Becton Dickinson). Os CSAs utilizados no estudo foram o conector de extensão multiplicador de acesso venoso *Luer Lock* (fêmea), de duas vias, neonatal (Polifix^®^, B. Braun), utilizado em CIVP, e o conector valvulado (Safeflow^®^, B. Braun), que pode ser utilizado em CIVP e em DAVC.

Os métodos de desinfecção testados correspondem à prática de desinfecção ativa de CSA em uma unidade de internação de hospital escola do sul do Brasil. O Incidin^®^ foi incluído no estudo por ser um produto utilizado na referida instituição. Ainda, sua aplicação ocorreu exclusivamente sobre superfícies externas dos dispositivos avaliados, não tendo contato direto com o lúmen do cateter ou com a corrente sanguínea do paciente devido ao tempo de secagem. Ressalta-se, também, que os resultados apresentados neste estudo ocorreram exclusivamente a partir de um modelo experimental *in vitro*, não implicando aplicação clínica nem recomendação de uso em dispositivos que entram em contato direto com pacientes, nem extrapolando as indicações regulatórias aprovadas pelo fabricante. Ainda, foram realizadas triplicatas técnicas e triplicatas biológicas, juntamente com os controles negativos e positivos (controles não tratados), para todos os experimentos. Ressalta-se que os experimentos ocorreram em condições estéreis, com uso de materiais estéreis.

### Realização do experimento: padronização, inoculação, tratamento e quantificação bacteriana

Para padronização do inóculo microbiano, a cepa de *Klebsiella pneumoniae*, armazenada a -20ºC, foi isolada e conservada em caldo *Brain Heart Infusion*, com período máximo de crescimento de 24 horas. Realizou-se repique bacteriano a cada nova repetição biológica, sendo preparada e cultivada conforme as recomendações do *Clinical and Laboratory Standards Institute* para “*Methods for Dilution Antimicrobial Susceptibility Test for Bacteria that Grow Aerobically*”^(14)^. Essa preparação permitiu a contagem das Unidades Formadoras de Colônia (UFC). Após a diluição inicial, utilizou-se uma suspensão 10^-6^, com média de 15 UFC/mL.

A suspensão bacteriana inicial foi preparada com turbidez correspondente ao tubo 0,5 da Escala McFarland (Newprov^®^) e conferida eletronicamente pelo DensiCHEK™ Kit (BioMililux), equivalendo a aproximadamente 1,5 x 10^8^ UFC/mL. Em seguida, preparou-se 1 mL da suspensão bacteriana de trabalho diluindo 100x a suspensão inicial (10 μL da suspensão bacteriana inicial de *Klebsiella pneumoniae* em 990 μL de solução salina estéril a 0,9%, pipetados em tubo cônico estéril do tipo Falcon 15 mL). A suspensão bacteriana de trabalho contou com aproximadamente 1,5 x 10^6^ UFC/mL. Os CSAs ficaram em contato com a suspensão de trabalho por um período de cinco minutos.

Cabe ressaltar que todos os experimentos incluíram controles positivos e negativos. Os controles positivos, também denominados controles não tratados, consistiram em CSAs que foram inoculados com a suspensão bacteriana e não submetidos a qualquer método de desinfecção, com a finalidade de quantificar os microrganismos aderidos aos conectores.

Os CSAs do controle negativo foram imersos em 1 mL de solução salina 0,9% por cinco minutos. Para cada dia de experimento, foram realizadas triplicatas técnicas e, em dias diferentes, triplicatas biológicas para garantir a reprodutibilidade e a confiabilidade do método. Cada CSA foi retirado do tubo cônico estéril do tipo Falcon com uma pinça estéril e posicionado em uma placa de Petri fechada e estéril para secagem por 60 minutos em estufa bacteriológica SL-101 (SOLAB) a 35°C ± 1. Após a secagem, cada um dos CSAs foi tratado com diferentes desinfetantes. O tratamento dos CSAs ocorreu de acordo com o manual do fabricante e seguindo as recomendações padrão da prática clínica, como das diretrizes da *Infusion Nursing Society* ^(2)^, da Agência Nacional de Vigilância Sanitária^(1)^ e do *Centers for Diseases Control and Prevention* ^(15)^.

O tratamento dos CSAs com os desinfetantes GE com etanol 70%, GNE com etanol 70% e GE com Incidin^®^ foi realizado da seguinte maneira: 1 mL do desinfetante foi pipetado na gaze. Em seguida, os conectores de extensão e conectores valvulados foram retirados da placa de Petri com uma pinça estéril. Após, foram realizadas fricções nos CSAs, girando a gaze no sentido horário e anti-horário por dez segundos (180º). No conector de extensão, a fricção com a gaze ocorreu sem a retirada da tampa pertencente ao conector. Foi permitida a secagem por cinco segundos dos dois CSAs.

Na desinfecção utilizando o dispositivo de uso único com IPA 70%, foram seguidas as recomendações do fabricante, sendo essas diferentes para conector de extensão e conector valvulado. Para o conector de extensão, foi retirada a tampa do mesmo e realizada a fricção com o dispositivo de uso único com IPA 70%, girando o mesmo no sentido horário e anti-horário oito vezes por dez segundos (180º). Com relação ao conector valvulado, o dispositivo de uso único com IPA 70% foi posicionado nele, e a fricção foi realizada da mesma forma que no conector de extensão, com secagem de cinco segundos. Todos os procedimentos de desinfecção foram realizados com a mão dominante de uma única pesquisadora, que conduziu o experimento *in vitro*, na tentativa de replicar a mesma força e técnica durante a realização dos experimentos.

Ressalta-se que o tempo de secagem de cinco segundos, adotado em todos os experimentos, foi escolhido por ser mais compatível com a prática clínica e por estar respaldado por evidências que demonstram redução significativa da contaminação bacteriana nesse tempo. Após exposição aos métodos de desinfecção, as amostras tratadas e os controles foram colocados em 2 mL de solução salina a 0,9%, em tubos cônicos estéreis do tipo Falcon de 15 mL, para permitir a recuperação dos microrganismos. Os tubos foram fechados com tampa estéril. Todas as amostras foram encaminhadas para serem vortexadas em equipamento agitador de tubos Phoenix (MOD AT56) por cinco minutos e, em seguida, passaram pelo processo de sonicação em um banho ultrassônico *UltraCleaner* 1400 (Unique), com frequência de 40 kHz, por dez minutos.

Na sequência, os tubos cônicos com os CSAs imersos passaram novamente pelo vórtex por cinco segundos para homogeneizar a amostra. Uma alíquota de 100 μL foi semeada em placas de Petri com *Tryptic Soy Agar* e distribuída em movimentos circulares com o espalhador de células formato T (Drigalski) até completa absorção do inóculo. Após incubação por 24 horas em estufa bacteriológica SL-101 (SOLAB) a uma temperatura de 35°C ± 1, foi realizada a contagem de UFC.

### Análise e tratamento dos dados

Para a análise de dados, foi realizada a comparação entre o número de UFCs entre os CSAs tratados e os controles positivos. Banco de dados foi desenvolvido no programa *Microsoft Excel* ^®^ 2016, para armazenamento, organização de dados e cálculo estatístico. Foram realizados dois testes, o teste não paramétrico de Kruskal-Wallis, com o objetivo de analisar e comparar grupos independentes, e a análise *post-hoc*, por meio do teste de Conover, para entender a interação entre os métodos de desinfecção. Foram utilizados os valores da mediana, pois se tratam de dados assimétricos, e foi adotado um nível de significância de p <0,05. O programa estatístico utilizado foi o MedCalc^®^, versão 19.1.7 (Bélgica).

## RESULTADOS

Foram realizados 84 experimentos no total, sendo 37 com os conectores de extensão e 47 com os conectores valvulados. Ao todo, foram incluídos 11 controles positivos, 12 controles negativos, 25 experimentos com tratamentos aplicados aos conectores de extensão e 36 experimentos com tratamentos nos conectores valvulados. Cabe ressaltar que *outliers* foram excluídos para não comprometer os experimentos, mantendo-se, no mínimo, duas duplicatas técnicas e duas duplicatas biológicas para a validação do experimento. Não houve crescimento bacteriano nos controles negativos, o que confirma a assepsia dos procedimentos e dos materiais utilizados.

Entre os CSAs, observou-se uma mediana de 377,5 UFCs, que se aderiram aos controles positivos dos conectores de extensão, e 236,0 UFCs, que se aderiram aos controles positivos dos conectores valvulados, indicando maior aderência da *Klebsiella pneumoniae* aos conectores de extensão em comparação aos conectores valvulados. Seguindo os protocolos de desinfecção estabelecidos na metodologia, verificou-se que todos os métodos testados foram eficazes na redução da carga bacteriana em ambos os CSAs.

Nos experimentos realizados com os conectores de extensão, a redução da carga bacteriana variou entre 69,01% e 96,56% (Tabela 1). Embora tenha havido variação nas taxas de redução entre os tratamentos com GE e GNE com etanol 70%, essa diferença não foi estatisticamente significativa.

**Tabela 1:** Eficácia de métodos de desinfecção ativa na redução da carga bacteriana de *Klebsiella pneumoniae* nos conectores de extensão. Florianópolis, SC, Brasil, 2025

Método de desinfecção ativa	Experimentos (n)	Mediana da UFC	Redução na contagem bacteriana (%)	Análise *post-hoc* (Conover)^*^ Diferença entre tratamentos (p<0,05)
(1) CNT	6	377,5	-	(2)(3)(4)(5)
(2) GE com etanol 70%	6	117	69,01	(1)
(3) GNE com etanol 70%	5	13	96,56	(1)
(4) GE com Incidin^®^	7	108	71,39	(1)
(5) Dispositivo de uso único IPA 70%	7	88	76,69	(1)
(6) CN	6	0	-	-

Legenda:

* Diferenças calculadas baseadas no teste de Kruskal-Wallis e análise post-hoc de Conover com graus de liberdade 4 e com p=0,004873; CNT - controle não tratado (positivo); CN - controle negativo, GE - gaze estéril; GNE - gaze não estéril; IPA - isopropanol; n - total de experimentos; UFC - Unidade Formadora de Colônia.

Dentro de todos os tratamentos utilizados nos conectores de extensão, o controle positivo foi diferente de todos os tratamentos de desinfecção. Porém, os tratamentos de desinfecção não apresentaram diferença significativa entre eles, mesmo apresentando variações na porcentagem de redução da carga bacteriana (Figura 1). Logo, todos os tratamentos mostraram-se eficazes, com o valor de significância de p=0,004873.

**Figura 1:** Comparação do grupo controle com os grupos de tratamento com relação ao número de Unidades Formadoras de Colônia de *Klebsiella pneumoniae* nos conectores de extensão. Florianópolis, SC, Brasil, 2025

Legenda: UFC - Unidade Formadora de Colônia; N - total de experimentos; CNT - controle não tratado (positivo); GE - gaze estéril; GNE - gaze não estéril; IPA - isopropanol; *Diferente do CNT.

Nos experimentos conduzidos nos conectores valvulados, foi possível perceber variação de redução da carga bacteriana mais homogênea entre os tratamentos do que nos conectores de extensão. Com isso, a redução da carga bacteriana variou entre 99,58% com o dispositivo de uso único com IPA 70% e 100% nos outros três tratamentos (p = 0,002960) (Tabela 2).

**Tabela 2:** Eficácia de métodos de desinfecção ativa na redução da carga bacteriana de *Klebsiella pneumoniae* nos conectores valvulados. Florianópolis, SC, Brasil, 2025

Método de desinfecção ativa	Experimentos (n)	Mediana da UFC	Redução na contagem bacteriana (%)	Análise *post-hoc* (Conover)^*^ Diferença entre tratamentos (p<0,05)
(1) CNT	5	236	-	(2)(3)(4)(5)
(2) GE com etanol 70%	9	0	100	(1)
(3) GNE com etanol 70%	9	0	100	(1)
(4) GE com Incidin^®^	9	0	100	(1)
(5) Dispositivo de uso único IPA 70%	9	1	99,58	(1)
(6) CN	6	0	-	-

Legenda:

* Diferenças calculadas baseadas no teste de Kruskal-Wallis e análise post-hoc de Conover com graus de liberdade 4, com p = 0,002960; CNT - controle não tratado (positivo); CN - controle negativo, GE - gaze estéril; GNE - gaze não estéril; IPA - isopropanol; n - total de experimentos; UFC - Unidade Formadora de Colônia.

A Figura 2 mostra a comparação entre os experimentos realizados nos conectores valvulados, sendo que todos apresentaram redução na carga bacteriana com relação ao controle positivo. Pode-se notar maior variação nos dados dos quartis emergentes do dispositivo de uso único com IPA 70%, sem que haja alteração na redução da carga bacteriana em comparação com o grupo não tratado. Todos os tratamentos foram eficazes na redução da carga bacteriana e não tiveram diferença entre si.

**Figura 2:** Comparação do grupo controle com os grupos de tratamento em relação à redução no número de Unidades Formadoras de Colônia de *Klebsiella pneumoniae* nos conectores valvulados. Florianópolis, SC, Brasil, 2025

Legenda: UFC - Unidade Formadora de Colônia; N - total de experimentos; CNT - controle não tratado (positivo); GE - gaze estéril; GNE - gaze não estéril; IPA - isopropanol; *Diferente do CNT.

## DISCUSSÃO

Foi possível comprovar a eficácia dos quatro métodos de desinfecção química ativa na redução da carga bacteriana de *Klebsiella pneumoniae* inoculada em conectores de extensão e conectores valvulados. Todos os tratamentos mostraram-se eficazes, com significância estatística na redução de UFCs, indicando potencial para mitigação do risco de ICS associada ao cateter.

Neste estudo, a desinfecção dos CSAs foi efetiva, com o uso do etanol 70%, Incidin^®^ e IPA 70%. Ensaio clínico randomizado (ECR), que comparou outros métodos de desinfecção em conectores valvulados, mostrou que não houve diferença na efetividade de *wipes* com IPA 70% e *wipes* com gluconato de clorexidina (CHG) 2% com IPA 70% para a desinfecção dos CSA. Por apresentarem baixo risco de alergia e baixo custo, o uso de IPA 70% por um período mínimo de cinco segundos de fricção foi recomendado^(16)^.

Em contrapartida, outro estudo aponta para a maior efetividade de desinfecção ativa com CHG 2% do que o uso de IPA 70%^(17)^. As diretrizes internacionais evoluíram com o avanço das evidências, enquanto as diretrizes anteriores incluíam iodopovidona entre as opções^(18)^. Atualmente, houve a exclusão dessa recomendação devido ao seu longo tempo de secagem (mais de seis minutos)^(2)^.

Cabe destacar que, neste estudo, foi utilizada gaze, a fim de refletir a realidade de muitos hospitais nacionais. Ainda, as gazes utilizadas foram estéreis e não estéreis, refletindo a prática diária da assistência de enfermagem. As não estéreis usadas neste estudo estavam armazenadas em locais limpos, como acontece nas unidades hospitalares. Estudo transversal realizado em unidades de internação de um hospital pediátrico no Brasil mostrou que 83,7% das desinfecções foram realizadas com GNE^(12)^. Porém, sabe-se que, em países como Estados Unidos da América, Austrália e países europeus, ocorre predominante o uso de *wipes* com IPA 70%, tampas de IPA 70%, *wipes* com CHG 2% e *wipes* com IPA 70%^(19)^.

Este estudo avaliou a aderência da *Klebsiella pneumoniae* ATCC^*®*^ 700603™ aos diferentes CSAs testados. Verificou-se que esta cepa bacteriana apresentou maior aderência ao conector de extensão do que no conector valvulado. Sugere-se que pode haver diferença entre o risco de ICS associada ao cateter para diferentes tipos de CSAs, sendo que as diferenças podem estar associadas ao *design*, à desinfecção utilizada e à forma de fabricação do CSA^(20)^, o que poderia ser uma hipótese para o achado.

Estudo que relacionou as ICS associadas ao Cateter Venoso Central (CVC) com o uso de diferentes CSA mostrou que o risco de infecção foi maior quando o paciente fazia uso de dânulas de três vias^(21)^. Sabe-se que as dânulas possuem *design* exterior similar ao dos conectores de extensão utilizados neste experimento. Por outro lado, o resultado do ECR realizado com pacientes cirúrgicos de UTI mostrou que o tipo de CSA não influenciou a taxa de ICS associadas ao CVC. Neste estudo, 23 pacientes do grupo de controle utilizaram dânulas, enquanto o grupo de intervenção, com 24 pacientes, utilizou o conector valvulado^(22)^.

Como mostrado em estudos anteriores, normalmente comparam-se dânulas de três vias e conectores valvulados^(21,23)^, devido ao uso mais comum em DAVC. No entanto, este estudo traz a comparação entre conectores de extensão, utilizados no cenário hospitalar brasileiro em CIVP, e conectores valvulados, utilizados também em CIVP, porém com maior frequência em DAVC.

Neste estudo, a fricção foi realizada por dez segundos, o que foi suficiente para reduzir a carga bacteriana. Em todos os experimentos, foi permitida a secagem por cinco segundos. Estudos anteriores comprovam a necessidade de tempos superiores a três segundos de fricção na porta de infusão intravenosa para uma diminuição significativa da contaminação bacteriana^(23)^. Outro estudo mostrou que a fricção foi efetiva em tempos de cinco, dez e 15 segundos^(16)^.

Nas diretrizes atuais, recomenda-se fricção de cinco a 15 segundos e secagem por cinco segundos quando utilizado o IPA 70%^(2)^. Logo, o tempo superior a cinco segundos já é eficaz para a redução da carga bacteriana, devendo ser levado em consideração juntamente com outros componentes da desinfecção, como tipo de desinfetante, tipo de CSA, técnica de fricção aplicada pelo profissional de enfermagem e tempo de secagem.

Revisão de escopo, que buscou identificar os métodos de desinfecção de CSAs, bem como a efetividade dos mesmos, reforça a importância de o profissional de enfermagem conhecer os agentes desinfectantes, assim como o tempo de fricção e de secagem. Porém, apesar dos vários métodos disponíveis, ainda não há consenso sobre qual seria o melhor a ser adotado na prática clínica^(24)^.

Estudo retrospectivo realizado em uma unidade de transplante renal, com realização de 900 transplantes nos últimos anos, evidenciou 86 casos de infecções por *Klebsiella pneumoniae* resistentes aos carbapenêmicos, sendo a corrente sanguínea o sítio mais frequente de infecção e com taxa de mortalidade geral de 36% em 30 dias^(25)^.

Outro estudo retrospectivo documental avaliou prontuários de pacientes que possuíam exames de cultura de bactérias positivas para *Klebsiella pneumoniae.* Evidenciou que a utilização de DAVC é um fator de risco para o desenvolvimento dessa infecção, estando presente em 18,8% dos casos, inclusive sendo registrado óbito em decorrência da infecção^(26)^. O aumento da resistência da *Klebsiella pneumoniae* aos antibióticos é preocupante^(25,26)^, exigindo que as instituições de saúde adotem medidas para reduzir as infecções causadas por esse bacilo. A desinfecção dos dispositivos venosos utilizados na TIV é uma ação importante a ser desempenhada pela equipe de enfermagem.

No contexto dos profissionais de saúde, a enfermagem, por ser uma profissão responsável pela manutenção dos acessos venosos e, consequentemente, dos CSAs, deve estar atenta a ações de assistência à saúde que podem levar a eventos adversos. Assim, a falta de evidências científicas, o desconhecimento dos enfermeiros acerca de determinadas ações associadas aos cateteres e a fragilidade na formação profissional com relação ao controle de infecções são riscos para a segurança do paciente^(27-29)^.

Afirma-se que a desinfecção é um dos passos para a prevenção e controle de ICS associada ao cateter por *Klebsiella pneumoniae* ^(30)^. Profissionais de saúde devem seguir os protocolos de prevenção de infecção e de redução da contaminação, como a higienização das mãos antes e após qualquer procedimento, e a correta desinfecção de superfícies que tenham tido contato com o paciente, como os CSAs^(31)^. Com isso, evidencia-se a necessidade de protocolos específicos para a prevenção de ICS associada ao cateter tanto periférico quanto central, no intuito de reduzir mortalidade e agravos decorrentes da assistência à saúde.

### Limitações do estudo

Como limitação deste estudo, cita-se a utilização de somente uma cepa bacteriana para a contaminação dos conectores de acessos venosos, o que não reflete a realidade da prática clínica. A utilização de cepa proveniente de bacterioteca permitiu maior controle experimental e reprodutibilidade dos resultados, embora não represente integralmente o comportamento de cepas clínicas multirresistentes, configurando-se como outra limitação do estudo. Ainda, o tamanho da amostra pode ter tido influência sobre os resultados, devido à retirada de *outliners*.

### Contribuições para as áreas da enfermagem e saúde

O estudo *in vitro* foi o primeiro passo para se conhecer o comportamento da *Klebsiella pneumoniae* mediante os agentes desinfetantes disponíveis nos hospitais brasileiros. Desse modo, é imprescindível que a enfermagem se aproprie desse conhecimento, para que sua prática assistencial esteja pautada em evidências científicas visando um cuidado seguro ao paciente, sem eventos adversos advindos da TIV. Além disso, com os resultados apresentados, é possível planejar ações de educação em saúde e/ou de formação continuada para disseminar e atualizar o conhecimento da equipe de enfermagem sobre a importância da desinfecção dos CSAs, bem como sobre o tempo de fricção e de secagem de cada produto, garantindo assim a efetividade do processo.

## CONCLUSÕES

Todos os métodos de desinfecção (tratamentos) foram eficazes para a redução da carga bacteriana de *Klebsiella pneumoniae* ATCC^®^ 700603™ inoculada em experimentos *in vitro.* Não houve diferença estatística entre os diferentes métodos de desinfecção ativa (GE com etanol 70%, GNE com etanol 70%, GE com Incidin^®^ e dispositivo de uso único com IPA 70%) como tratamentos em conectores de extensão e em conectores valvulados. Portanto, os resultados demonstraram que a fricção por dez segundos com uma das três soluções desinfetantes (etanol 70%, Incidin^®^ ou IPA 70%), seguida de secagem dos CSAs por cinco segundos, é eficaz na redução da carga bacteriana de *Klebsiella pneumoniae.*

Esses achados são relevantes para a prática clínica de enfermagem, pois permitem conhecer métodos de desinfecção efetivos de CSAs, que podem levar à redução das taxas de ICS associadas ao cateter em hospitais para pacientes que fazem uso de CIVP e DAVC. Esse é o primeiro passo para a identificação de práticas seguras de desinfecção de CSAs, sendo que deve ser seguida pela realização de ECR, a fim de confirmar os achados experimentais e construir protocolos a serem seguidos pelos profissionais de saúde.

Revela-se importante realizar repetições de experimentos com o uso de GE e GNE, a fim de verificar a diferença entre as apresentações, mesmo tendo sido considerada uma variação experimental sem significância estatística. Além disso, os custos e os benefícios devem ser levados em consideração para a escolha do tipo de desinfetante e de desinfecção utilizada em hospitais brasileiros.

## Data Availability

Os dados de pesquisa disponíveis estão no corpo do artigo.
